# Allele-Specific Behavior of Molecular Networks: Understanding Small-Molecule Drug Response in Yeast

**DOI:** 10.1371/journal.pone.0053581

**Published:** 2013-01-04

**Authors:** Fan Zhang, Bo Gao, Liangde Xu, Chunquan Li, Dapeng Hao, Shaojun Zhang, Meng Zhou, Fei Su, Xi Chen, Hui Zhi, Xia Li

**Affiliations:** College of Bioinformatics Science and Technology and The Second Affiliated Hospital, Harbin Medical University, Harbin, P. R. China; Virginia Tech, United States of America

## Abstract

The study of systems genetics is changing the way the genetic and molecular basis of phenotypic variation, such as disease susceptibility and drug response, is being analyzed. Moreover, systems genetics aids in the translation of insights from systems biology into genetics. The use of systems genetics enables greater attention to be focused on the potential impact of genetic perturbations on the molecular states of networks that in turn affects complex traits. In this study, we developed models to detect allele-specific perturbations on interactions, in which a genetic locus with alternative alleles exerted a differing influence on an interaction. We utilized the models to investigate the dynamic behavior of an integrated molecular network undergoing genetic perturbations in yeast. Our results revealed the complexity of regulatory relationships between genetic loci and networks, in which different genetic loci perturb specific network modules. In addition, significant within-module functional coherence was found. We then used the network perturbation model to elucidate the underlying molecular mechanisms of individual differences in response to 100 diverse small molecule drugs. As a result, we identified sub-networks in the integrated network that responded to variations in DNA associated with response to diverse compounds and were significantly enriched for known drug targets. Literature mining results provided strong independent evidence for the effectiveness of these genetic perturbing networks in the elucidation of small-molecule responses in yeast.

## Introduction

Elucidation of the molecular and genetic basis of phenotypic variation has been a longstanding goal in genetics, including all aspects of morphology, physiology, behavior, disease susceptibility, and drug response. Genome-wide association studies (GWAS) mapping quantitative trait loci (QTLs) have enabled the identification of regions of the genome in which genetic variations are associated with phenotypic variation. However, the computational understanding of the biological mechanisms underlying genetic variations remains unclear. Numerous previous studies have dissected the downstream effects of genetic perturbations on RNA intermediates, proteins, metabolites and other molecular endophenotypes [1], [2], [3], [4], [5]. Whole-genome expression QTL (eQTL) analysis in yeast, mice, and humans has demonstrated that gene expression traits are highly inheritable and exhibit surprisingly complex underlying genetic architecture [6], [7], [8]. By layering gene expression phenotypes as intermediate phenotypes, many studies have combined eQTL and disease GWAS to identify causal relationships between genes and disease (reviewed by Ertekin-Taner [5]). Therefore, further elucidation of changes in molecular states that directly respond to changes in DNA could potentially fill in the information gaps left by GWAS, and serves as an excellent first step to understanding the drivers of a complex phenotype.

However, most proteins perform their functions through interactions with other proteins, or as part of biochemical pathways and networks. Thus, some genes may respond as groups due to their membership in networks. As an alternative to the studies assuming that genes act independently, certain previous studies have utilized an effective approach that assessed higher-order a priori defined gene network responses caused by genetic variation [9], [10], [11], [12], [13], [14], [15], [16]. However, these studies only considered the variation of the synthetic expression of genes in a priori defined networks, and failed to model how genetic variations are mediated by a network of molecular interactions in the cell. In addition, the association of these networks that are affected by genetic variations with the resultant phenotypic variation remains poorly understood.

The use of systems genetics is changing the future of genetics. Moreover, it is a corollary to the basic insight of systems biology in that most complex traits of living things are properties generated by dynamic networks of interacting genes and molecules [17], [18]. In recent years, our ability to interpret the phenotypic variation in model systems, and ultimately in humans, has benefited from systems genetics approaches [17], [18], [19], [20], [21], [22], [23]. Chen et al. [21] constructed co-expression networks through the combination of gene expression and genotype data, and uncovered components of the co-expression networks that respond to genetic variations associated with disease-associated traits. This study confirmed that complex traits, such as obesity, are potentially emergent properties of molecular networks modulated by complex genetic loci and environmental factors. Zhong et al. [23] proposed a model to describe the effect of disease-causing mutations in Mendelian disorders on systems or interactome properties. Equipped with the tools emerging from the genomics revolution, the study of the effects of genetic perturbations on molecular networks will serve as an important aspect of systems genetics research.

Various biochemical or biophysical interaction(s) are the building blocks of biological functions and processes and are also the basic units of molecular networks; thus, it is important to investigate the effects of genetic variations on molecular interactions. Recently, several reports have explored the effects of genetic variations on protein-DNA interactions [24] or protein-protein interactions [23] from the perspective of the structural properties of physical binding. In this study, through integrating gene expression data, genotype data, and molecular interactions, we developed models to detect two types of allele-specific perturbations on interactions, termed allele-specific co-perturbation (ASCP) and allele-specific dys-perturbation (ASDP). In both cases, a genetic locus exerted a different influence on the transcriptional program of interactions for cases in which the locus exhibited distinct (alternative) alleles.

In this study, using a well-controlled system, we aimed to investigate how molecular networks respond to DNA perturbations based on the interaction perturbation models (ASCP and ASDP), and thus, drive variations in physiological states associated with phenotypic variation. Therefore, we used a panel of 112 genotyped and expression-profiled yeast strains [6] which were also treated with a collection of 100 diverse compounds, termed small-molecule perturbagens (SMPs) [25], to investigate the effect of genetic variations (such as Single Nucleotide Polymorphisms, SNPs) on the dynamic behavior of an integrated interactome of yeast, and to elucidate the molecular mechanisms underlying individual differences in response to small-molecule drugs in yeast based on networks that exhibit genetic perturbation.

## Results

### EQTL Mapping

We performed eQTL mapping using gene expression and SNP genotyping data on 112 segregants generated in a cross between laboratory (BY) and wild (RM) strains of *Saccharomyces cerevisiae* [6]. We sought to identify an initial set of potential associations between eQTLs and their target genes for further analysis. In order to determine the statistical significance more accurately, we merged adjacent markers that exhibited the same genotype profile to obtain a total of 1118 representative markers and selected 4500 transcripts with significantly high heritability (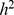
>0.669 at a false discovery rate, FDR of 0.05) (see Materials and Methods). Finally, we performed linkage calculations between 1118 genetic markers and 4500 transcript levels using the Student’s *t*-test, and assessed significance via permutations (see eQTL mapping section in Materials and Methods for algorithmic details). As a result, we detected 30,793 associated transcript-locus pairs (FDR<0.05). There were 3175 transcripts (3141 ORFs) linked to at least one QTL and an average of 9.7 QTLs per transcript. A total of 1112 markers were linked to at least one target transcript. To determine whether the loci identified by linkage act in cis or in trans, we investigated transcripts whose levels were linked to markers within 10 kb of their own gene. We found that 656 (21%) of the 3175 transcripts fell into the cis-acting category and most expression differences mapped to trans-acting loci. Our findings are in concordance with other reports in which trans-acting loci appear to be responsible for most differences in gene expression between yeast strains [26], [27].

### Determining Allele-specific Perturbation of Interactions

To assess how variations at the genetic level that affected gene expression levels (eQTLs) would alter molecular interaction states in the cell, we introduced allele-specific perturbation of interaction; we defined this as: a genetic locus with alternative alleles that exerts a different influence on an interaction. Two models were developed to detect the allele-specific behavior of interactions: allele-specific co-perturbation (ASCP) and allele-specific dys-perturbation (ASDP) (a detailed description can be found in the Materials and Methods section). ASCP denotes that an eQTL synchronously regulated the expression of two interacting genes. ASDP denotes that an eQTL triggered significant changes in expression correlation between two interacting genes. Each interaction in an integrated interactome, containing protein-protein interactions (PPI), protein-DNA interactions (PDI), kinase-protein interactions (KPI), and enzyme-enzyme interactions (EEI) (a detailed description of the interaction types can be found in the Materials and Methods section), was then analyzed to determine which demonstrated allele-specific behavior based on the two models. As a result, we identified 10440 ASCP relationships between 408 eQTLs and 2184 interactions, and 5774 ASDP relationships between 758 eQTLs and 2416 interactions (*P*<0.001, FDR<0.082). A total of 70.86% (788/1112) of the eQTLs perturbed at least one interaction. In addition, we analyzed the cis-acting or trans-acting regulation between the genetic loci and their perturbed ASCP and ASDP interactions. Five different conditions were investigated and detailed information is shown in Table S1. Interestingly, we found that many interacting genes in ASCP and ASDP interactions are located on the same chromosomes, even located adjacent to their regulators (eQTLs). This phenomenon may reflect the linear arrangement and aggregation of yeast genes on the same chromosomes which are required for coordinate expression of genes involved in related metabolic or regulatory pathways [28], [29], [30]. In addition, among the 3141 target genes associated with at least one eQTL, approximately 65% exhibited allele-specific perturbation of interactions with their partners in the interactome. It has been suggested that for a considerable portion of target genes, the eQTLs that disturbed their expression levels would also cause a degree of dynamic variation in their local network. Various biochemical or biophysical interaction(s) are the basic units of a molecular network and also the building blocks of biological functions and processes; thus, exploration of the allele-specific behavior of interactions makes it possible to test the downstream effects of genetic perturbations on molecular networks.

### Comparison of the Effect of ASCP and ASDP on Networks

Table 1 shows the overall statistics of perturbed interactions detected by ASCP and ASDP models. Firstly, we found that 13.53% (4443/32831) of the interactions between target genes (of eQTLs) and their partners in the integrated network are perturbed by at least one eQTL. This finding indicates that many of the interactions (∼86%) may behave consistently across individuals with different genotypes and represent a cellular network ‘backbone’. Secondly, we found that PPIs in complexes exhibited ASCP more frequently. When annotated to 305 protein complexes obtained from the CYC database [31], 162 of the 939 (17.3%) ASCP PPIs were found to be in the same complexes, whereas only 56 of the 819 (6.8%) ASDP PPIs were located in the same complexes (Fisher’s exact test, *P* = 1.184e–11). For PDIs, regulatory relationships between TFs (TF-TF) were also observed more frequently in ASCP (Fisher’s exact test, *P* = 0.03809), whereas phosphorylation events between two kinases (K-K) were observed more frequently in ASDP (*P* = 0.03245) (Figure S1A).

**Table 1:** Summary of the global effect of ASCP and ASDP on the four studied molecular interaction networks and the integrated interactome.

NETWORK	Edges	ASCP	ASDP	Both	Percentage
PPI	12067	939	819	1703	14.11%
PDI	6815	524	616	1056	15.50%
KPI	13532	550	842	1336	9.87%
EEI	1912	261	237	421	22.02%
COMBINED	32831	2184	2416	4443	13.53%

The second column denotes the total number of interactions detected for allele-specific behavior. The third and the fourth column represent the number of ASCP and ASDP interactions detected respectively. The fifth column represents the total number of perturbed interactions when considering both ASCP and ASDP, while the proportion they occupy in all interactions tested is listed in the sixth column.

In order to facilitate analysis, we constructed two networks called CPN and DPN that contained all of the ASCP and ASDP interactions, respectively. As shown in Figure S1B, most interactions in CPN are connected and form a large connecting component that contains ∼90% of all nodes; the same is observed in DPN (∼93% of all nodes). These results indicate that the transmission of the effect of genetic variations is mediated by network continuity. By merging these two types of perturbed interactions, a larger system is formed, thus, the coordination of these two disturbance effects is evident. We evaluated the degree distribution of genes and observed a power-law in both CPN and DPN (Figure S1C). This suggests that both models of genetic perturbing networks display scale-free characteristics [32]. CPN and DPN shared approximately 39.8% (910/2286) proteins but only approximately 3.53% edges, indicating that a large quantity of interactions is rewired between CPN and DPN (Figure S1D). Thus, these two models represent various genetic perturbing actions on a molecular network. Hence, ASCP and ASDP could potentially reveal the condition-specific dynamic information hidden among otherwise common static interactions.

### The Interactome Exhibits a Modular Changing Pattern Under Allele-specific Context

**Figure 1:**
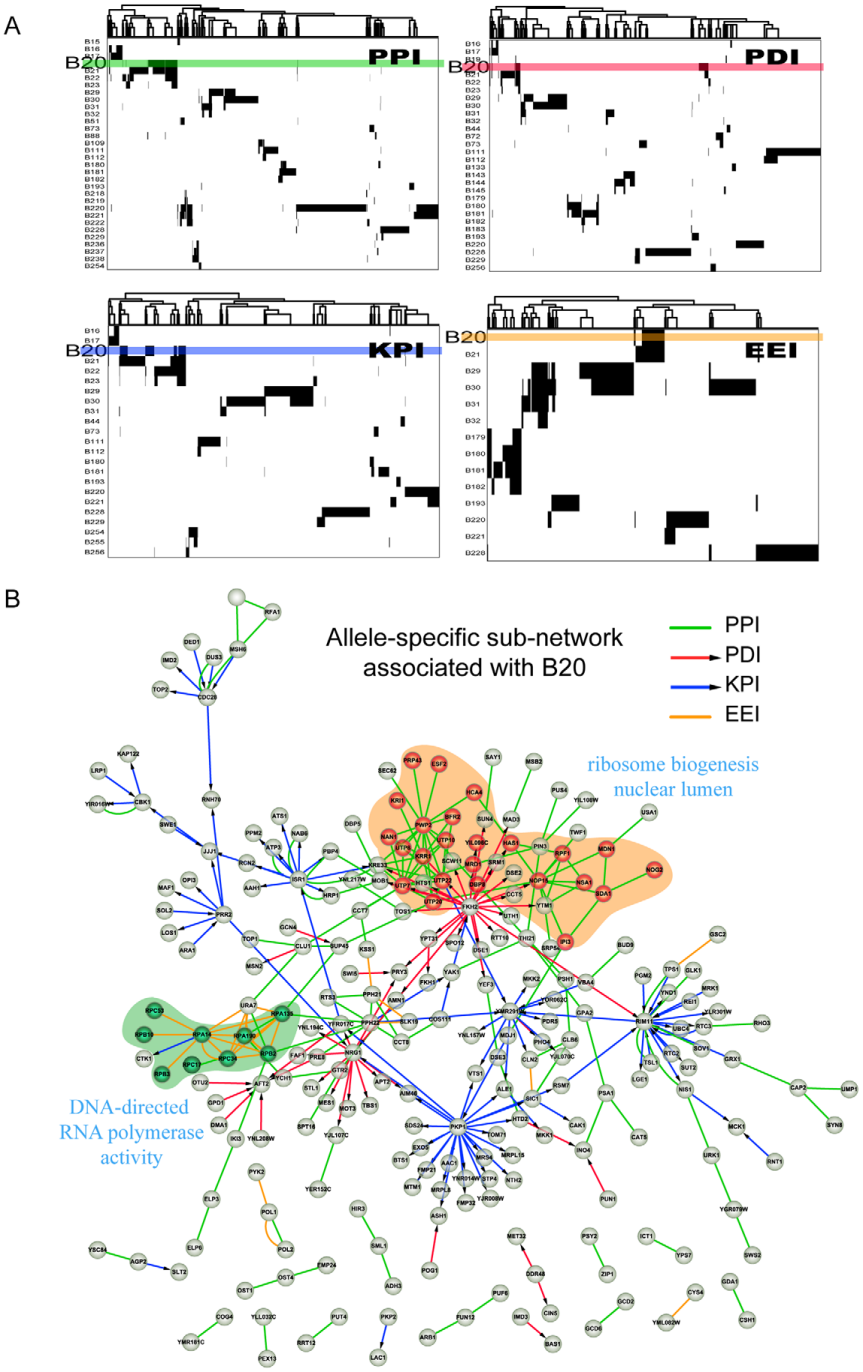
The changing pattern of molecular networks under allele-specific context determined by LD blocks. (A) Hierarchical clustering on the perturbed interactions based on the association matrix between blocks and their perturbing interactions in PPI, PDI, KPI, and EEI networks, respectively, in which rows represent blocks and columns represent interactions. In each of the four sub-matrices, blocks associated with less than ten interactions are filtered out. The columns are reordered according to hierarchical clustering. The rows colored correspond to B20. (B) The allele-specific sub-network associated with B20. The sub-network is composed of 142 PPIs, 54 PDIs, 93 KPIs, and 26 EEIs, which are marked with green, red, blue and orange, respectively. Nodes marked with red are proteins constituting a GO cellular component (nuclear lumen), and PPIs among these genes are regulated by B20. Nodes marked with green are enzymes from a KEGG pathway (DNA-directed RNA polymerase activity), and their EEIs are regulated by B20.

As discussed above, when genetic variations occurred on DNA sequences, some gene expression levels are affected (eQTL mapping), and cause the alteration of interaction states (allele-specific perturbation on interactions). Next, dynamic changes in networks would be triggered. In this study, we introduced an allele-specific context to capture the changing patterns of a network from one allele state to another. Due to the linkage disequilibrium (LD) of adjacent eQTLs, we identified 263 haplotype LD blocks (B1–B263) defined by the non-random association of genetic variants at two or more loci using Haploview software [33]. We then assembled all ASCP and ASDP interactions associated with the eQTLs located in the same block region. As a result, we found 246 blocks associated with at least one interaction; of these, 180 were associated with at least three edges. Next, we built an association matrix that represented the allele-specific perturbation relationships among all of the blocks and their perturbed interactions, and performed hierarchical clustering on the perturbed interactions. Figure 1A shows the clustering results of four sub-matrices extracted from the overall association matrix. These sub-matrices represent the associations between 32 blocks and 1446 PPIs, 30 blocks and 937 PDIs, 23 blocks and 1078 KPIs, and 14 blocks and 366 EEIs, respectively. Our findings indicated that for all four types of networks, each block primarily regulated a small portion of the network and that distinct parts of the network are primarily modulated by a few particular blocks. Thus, a complicated combination is evident, in terms of both target specificity and cooperativity of blocks. Moreover, the overall integrated network presented the same changing pattern under an allele-specific context (Figure S2).

**Figure 2:**
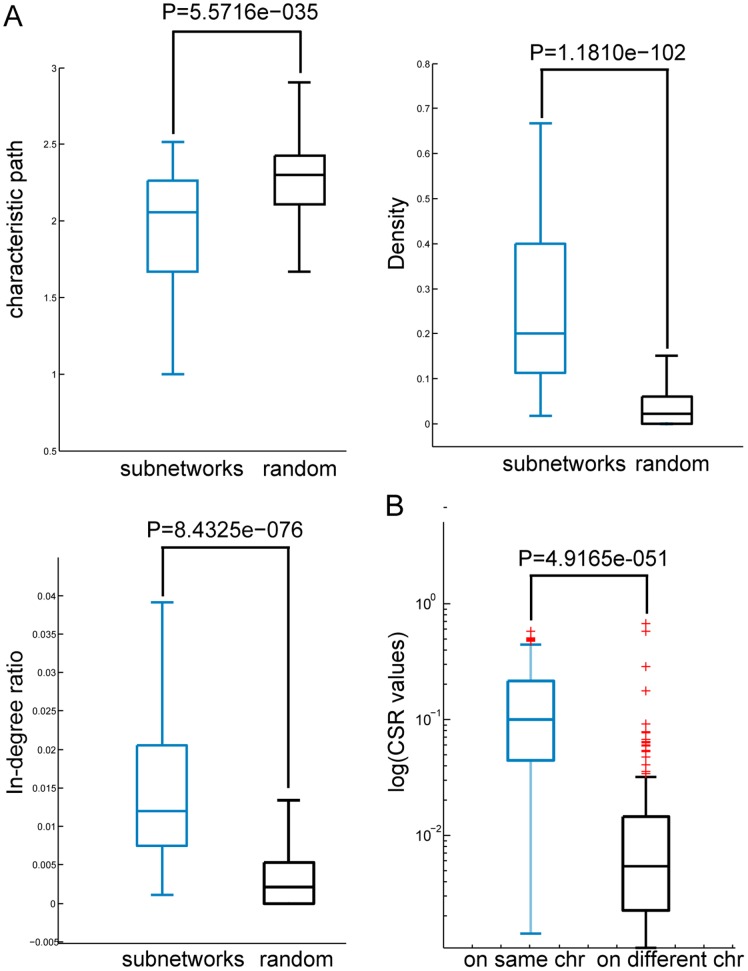
A summary of the sub-graph properties of the sub-networks associated with LD blocks. (A) The characteristic path length among genes in the same allele-specific sub-networks is significantly shorter compared to a randomization test. Moreover, the density and in-degree ratio of these sub-networks are significantly greater compared to a randomization test. Boxes of light color represent the distribution of the sub-graph properties (characteristic path length, density, in-degree ratio) of allele-specific sub-networks, and the black boxes correspond to random ones. (B) The panel shows the difference in CSR values between block pairs on the same chromosomes and on different chromosomes. *P*-values are calculated using the Wilcoxon rank-sum test.

To further analyze the role that individual blocks play in the regulation of the integrated network, we mapped an allele-specific sub-network for each block. Each sub-network corresponded to a minimal component in the original integrated network that contained all the genes consisting of the interactions associated with a block. It is guaranteed that all of the interactions perturbed by the block are located in this network component. We investigated the sub-graph properties of these sub-networks to determine their topological features in the integrated network. A summary of the results is listed in Table 2 and Figure 2A. Computer simulation results demonstrated that the topological features of these sub-networks were significantly different from randomized networks (details of the randomization test are provided in Materials and Methods). The results indicated that the characteristic path length between genes in the same sub-network was significantly shorter, and the average density of these sub-networks was significantly greater. Moreover, the average in-degree ratio of the sub-networks was significantly higher, indicating that proteins composing the allele-specific sub-network jointly exhibit significant modularity in the integrated network. The in-degree ratio is defined as the ratio (R) of in-degree to out-degree of a sub-network, in which “in-degree” represents the number of its connections within the sub-network, and “out-degree” represents its connections outside sub-network [34]. In total, 162 blocks (89.5% of 180 blocks investigated) were demonstrated to perturb interactions located in a module of the integrated network (FDR<0.05). In other words, the alteration of allele state of a block potentially leads to changes inside a network module. In addition, modules in the integrated network are composite modules; thus elucidation of the complex relationships underlying multiple biological interaction types will further the understanding of complex cell processes. For example, we found that B20 on chromosome II regulates 318 interactions among 246 genes, including 93 KPIs, 54 PDIs, 142 PPIs, and 26 EEIs (Figure 1B). Most interactions (92%) perturbed by B20 were connecting components gathered in a network module, moreover, various types of interactions were gathered in certain areas; the PPIs in the area marked by orange constitute a cellular component called nuclear lumen, and the EEIs in the area marked by green are derived from a pathway that controls DNA-directed RNA polymerase activity. These findings suggest that the information exchange and collaboration occurs among multi-layer molecular networks.

**Table 2:** Summary of topological properties of the allele-specific sub-networks and their Z-scores.

	All	ASN			Random
	Mean	Mean	<Z>	*p*-value	Mean
Char.path length	2.857	2.0548	−12.34	5.57e–035	4.27
Density	0.004	0.2226	21.51	1.18e–102	0.046
In-degree ratio	N.A.	0.0175	18.42	8.43e–076	0.0065

Topological properties of the yeast integrated network (All), the allele-specific sub-networks associated with blocks (ASN), and the random sub-networks (Random). The average *Z*-scores and the *p*-values for the observed properties of allele-specific sub-networks are also listed, respectively. N.A. not applicable.

### Proximal Blocks Regulating Similar Network Components

It has been observed previously that different chromosomal regions (LD blocks) regulate common network modules together. Here, we found a total of 423 pairs of blocks associated with overlapping allele-specific sub-networks. We introduced a score termed Component Share Ratio (CSR) to measure the degree of overlap between two network components regulated by two distinct blocks (Materials and Methods). This value ranges from 0 to 1; the higher the score, the more overlap between two network components, with 1 indicating that two blocks regulate identical network components.

We divided 423 pairs of blocks that exhibited a CSR value into two groups: 211 block pairs were located on the same chromosome and 212 on different chromosomes. Next, we calculated the significance of the differences between the two groups and found that block pairs on the same chromosome exhibited significantly higher CSR values (log-transformed) compared to block pairs on different chromosomes (*P* = 4.9165e-051, Figure 2B). In this study, a higher CSR value reflects a higher gene composition and function similarity between two blocks’ perturbed network modules. It is observed that the regulator-pairs (LD block-pairs) on the same chromosome have much higher CSR values. Moreover, the network modules regulated by the blocks on the same chromosomes, especially on adjacent regions, are enriched in metabolic processes, signaling pathways and enzyme catalyzed processes. It is suggested that LD blocks on the same chromosomes, especially adjacent blocks tend to involve in the regulation of common or correlated functions. This phenomenon may reflect the coordinated regulation requirement of genes involved in related metabolic or regulatory processes [29], [30]. In addition, some network modules perturbed by block-pairs on separate chromosomes also have high CSR values; this may imply the universality and genetic complexity of the yeast genome in the regulation of molecular networks.

### Biological Features of the Allele-specific Sub-networks Associated with Blocks

We evaluated the biological features of these allele-specific sub-networks. We classified 2286 genes from all of the sub-networks into 85 functionally related gene groups (or classes) (F1–F85) with enrichment score > = 1.3 (equivalent to non-log scale 0.05) using the Functional Classification Tool provided by DAVID [35], [36], with 833 genes not included in any group. Next, we performed functional enrichment analysis using hypergeometric distribution and detected 158 enrichment relationships (FDR<0.05 *P*<0.0039) between 87 sub-networks and 48 functional groups. Using blocks to represent their associated sub-networks, these enrichment relationships are presented as a bipartite graph between 87 blocks (triangle) and 48 functional groups (circle) (Figure S3).

**Figure 3:**
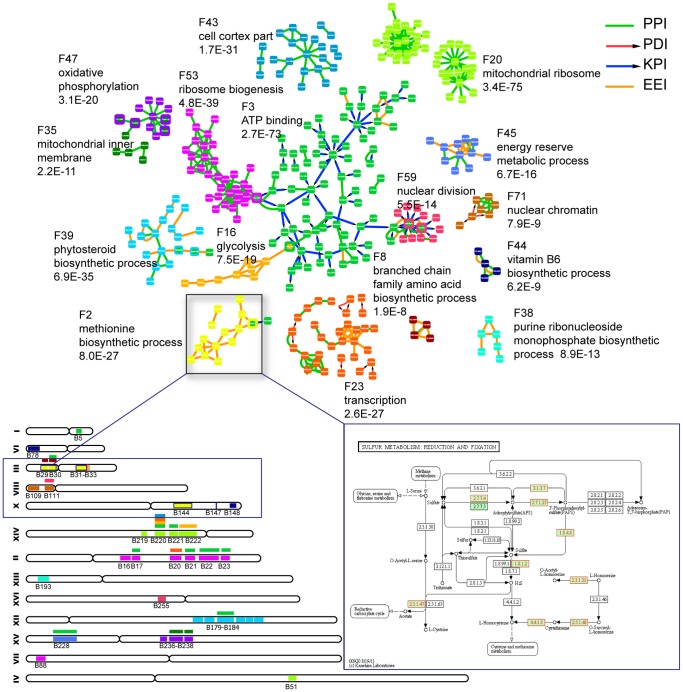
Biological features of the allele-specific sub-networks associated with LD blocks. A reconstructed network created by combining all of the allele-specific perturbed interactions associated with the 36 blocks and belonging to their enriched 16 functional groups. The genes belonging to different functional groups are marked with the same colors as in figure S3; also, chromosome blocks are marked using the same colors as their regulating functional classes. Functional annotation was conducted for each dyed gene module using DAVID based on GO and KEGG, and the most enriched functional item covering total genes (100%), along with its significance level (p-value), is presented near the dyed gene module. The gene module marked yellow is mainly associated with blocks on chromosome III (B29–B31) and X (B144); the lower right corner is a schematic representation of the metabolic pathway: sulfur metabolism, reduction and fixation, and nine enzymes in the module annotated to the pathway are marked with red rectangles.

To create an intuitive display of the role that different chromosome segments (LD blocks) played in molecular network regulation or perturbation, we filtered out 57 relationships between 36 blocks and 16 functional groups (57 red edges in Figure S3). For each enrichment relationship between a block and a functional group, we extracted the perturbed interactions associated with the block and belonging to the enriched functional group. We re-constructed a network, shown in Figure 3, by combining all of the interactions extracted; the functional modular regulation mode of genetic variations on the multi-layer integrated molecular network can be seen. Different chromosomal regions (blocks) that control or affect different parts or modules of the network exhibit intuitive biological features and trigger the modification of specific cellular functions. For instance, blocks on chromosome III (B29–B31) and X (B144) (yellow rectangles) are associated with a network module containing 14 enzymes (black box). This module is generally annotated as the “methionine biosynthetic process” (enrichment significance: *P* = 8.0E–27), and nine enzymes are located in the metabolism pathway-“sulfur metabolism: reduction and fixation”. Moreover, some functional groupings are found to be under the control of common blocks. For example, blocks on chromosome XIV (B219–B222) are associated with the cellular component “mitochondrial ribosome” and also regulate other functional classes, such as glycolysis, ATP binding, transcription, and the cell cortex. The mitochondrial ribosome is responsible for the biosynthesis of protein components crucial to the generation of ATP in the eukaryotic cell. Similar cases are the perturbation of blocks B237–B238 (simultaneously influencing oxidative phosphorylation and mitochondrial inner membrane), B111 (nuclear chromatin and nuclear division), and B29–B30 (methionine biosynthetic process and branched chain family amino acid biosynthetic processes). Most of the joint perturbations by blocks reflect the coordination among different biological processes, molecular functions, and cellular components in a certain cellular function, and ATP (ATP binding) is one of the most generic sites (targeted by B179–B184, B228, etc.) in these processes (Figure 3).

### Application in Understanding Small-molecule Drug Response in Yeast

**Figure 4:**
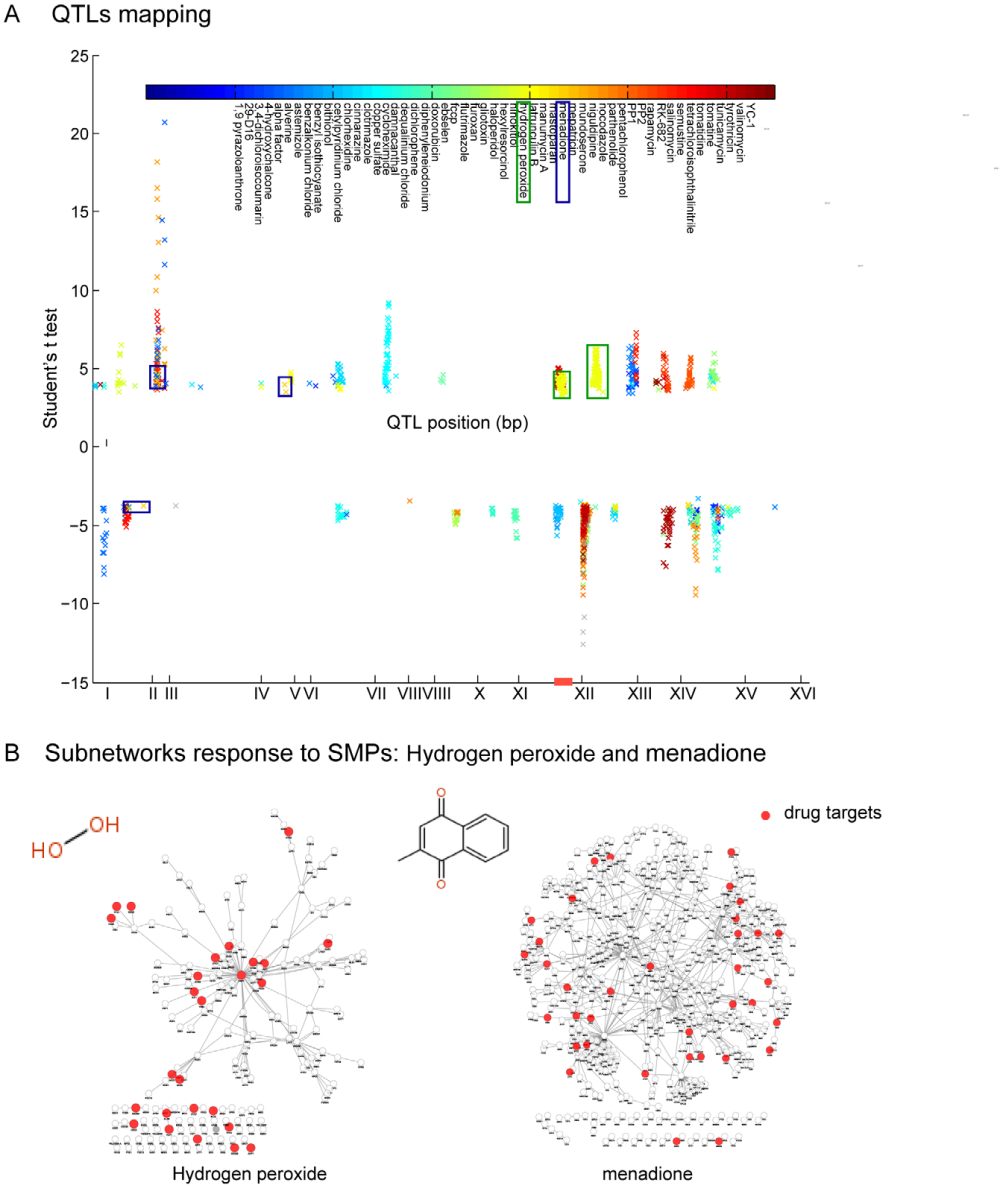
The QTLs distribution and drug targets enrichment of SMPs. (A) The QTLs distribution along the whole genome for 53 small-molecule drugs. QTLs associated with different SMPs are marked with various colors. (B) The sub-networks responding to the SMP response of hydrogen peroxide and menadione. Red nodes are known drug targets included in the STITCH database.

**Figure 5:**
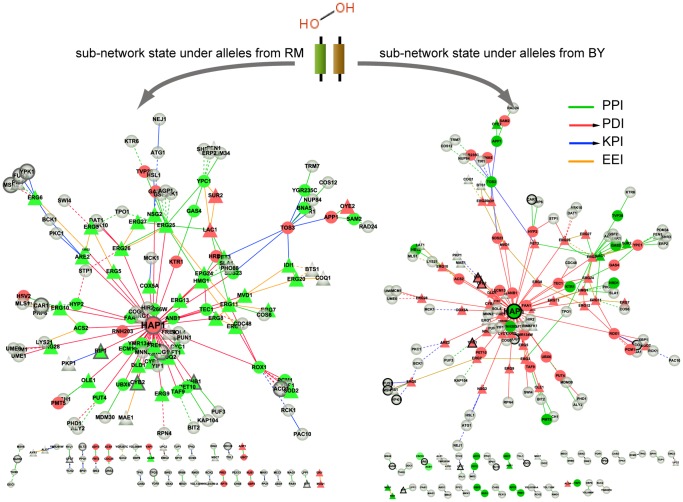
The state of the sub-networks in response to hydrogen peroxide in segregants inherited alleles from RM and BY. Solid edges indicate correlated expression between proteins in one allele state, whereas that organization is lost in another allele state (dotted line). Edge colors represent diverse interaction types between proteins, whereas node colors represent changes in gene expression between wild and mutant groups; green denotes down-regulation and red denotes up-regulation. Triangle nodes present genes annotated in ‘ergosterol metabolic process’, ‘lipid biosynthetic process’, and ‘oxidation reduction’. Nodes with dark edges are known drug targets of hydrogen peroxide based on Stitch2 database.

We utilized the model of genetic perturbing networks discussed above to gain a systematic understanding of small molecule perturbagen (SMP) response in genetically distinct yeast individuals. Firstly, we conducted linkage analysis between 324 phenotypes (segregant final yields of 100 SMPs at multiple time points and concentrations) and 2,956 genetic markers previously genotyped in the segregants [6], [25]. We identified 1470 QTLs with an absolute value *t* statistic score >3.28 (FDR<0.05) using a method identical to eQTL mapping, and 201 QTLs were in concordance with previous studies [25], covering nearly 92% of the original result (219 QTLs with a logarithm of the odds (lod) score
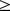
4). Because all time points and concentrations of each SMP were used as independent phenotypes, we combined QTLs associated with the same SMP but different concentrations and time points, and obtained QTLs for 92 SMPs. The position distribution for the QTLs along the genome is shown in Figure 4A. Furthermore, for 91 SMPs, we obtained a sub-network perturbed by QTLs associated with each SMP response trait. Among these SMP response associated sub-networks, 16 were significantly enriched for known drug targets based on the entire chemical–protein interactions set obtained from the STITCH database [37], such as hydrogen peroxide (H2O2) (*P* = 5.70E–06) and menadione (*P* = 3.50E–07) (Figure 4B and Table 3). In total, 22 high-confidence (SCORE
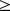
700) yeast chemical–protein interactions were found in SMP associated networks, such as hydrogen peroxide, which targets the proteins (*SOD2*, *CYB2*); rapamycin, which targets the proteins (*TOR2*, *LST8*); and staurosporine, which targets the protein (*YPL236C*). This suggests that these sub-networks potentially reflect the molecular mechanisms of the drug action. Taking hydrogen peroxide (*H2O2*) as an example, the sub-network response to it is highly enriched for the GO biological processes ‘ergosterol metabolic process’, ‘lipid biosynthetic process’, and ‘oxidation reduction’ (*P*<5.7E–24, *P*<2.1E–19, and *P*<2.1E–7). The expression levels of the majority of the genes annotated to these GO terms were down-regulated in response to H2O2 in wild type (Figure 5, green triangles in left branch under allele state from RM). This observation is in concordance with the report by Pedroso and Folmer [38], [39] in which the adaptation to H2O2 was observed to repress the expression of genes (*ERG3*, *ERG6*, *ERG7*, *ERG25*, *FEN1*) coding for enzymes involved in both ergosterol biosynthesis and lipid metabolism in wild strains. The QTLs associated with H2O2 are located on chromosome XII and chromosome XIII. A LD block on chromosome XII contains the candidate gene *HAP1* (*YLR256W*), which encodes a heme-responsive zinc-finger transcription factor whose expression varies according to oxygen levels in the cell, thus regulating oxygen dependent gene expression. As shown in Figure 5, *HAP1* serves as an important regulator and it regulates most of the proteins associated with H2O2 response. It has been previously reported that *HAP1* with a Ty1 insertion induces expression changes of sterol biosynthesis genes [40]. Additionally, it is a known drug target of H2O2 and is included in the STITCH database. By utilizing our proposed model, we were able to identify molecular network components that respond to small-molecule drugs; this provided insight into how networks states changed from wild-type to mutant-type when responding to SMPs (Figure 5). Characterizing molecular network states that underlie complex traits, such as drug response, potentially provides a comprehensive view, which in turn could potentially lead to the direct identification of genes or pathways underlying drug response processes and aid in the elucidation of the functional roles of these genes with respect to drug response. Thus, based on the modeling of genetic perturbing networks that respond to SMP, we investigated the potential biological application of drug function and drug target prediction, and investigated whether our results would uncover new and possibly non-intuitive relationships between biochemical pathways.

**Table 3:** Summary of enrichment analysis of known drug targets in the sub-networks associated with 16 small-molecule drug responses.

Compound response	m	k	x	N	*p*	q (<0.05)
hexylresorcinol	1	96	1	6063	0	0
rapamycin	265	966	76	5799	2.59E–08	2.59E–07
menadione	149	637	36	5915	3.50E–07	2.10E–06
staurosporine	123	755	35	5941	4.21E–07	2.10E–06
cycloheximide	188	365	28	5876	2.04E–06	8.15E–06
hydrogenperoxide	314	205	26	5750	5.70E–06	1.90E–05
anisomycin	33	450	10	6031	1.44E–05	4.11E–05
ascomycin	10	1007	6	6054	0.000257504	0.000643761
tamoxifen	33	147	4	6031	0.001073139	0.002384754
doxorubicin	109	123	7	5955	0.001565578	0.003131156
cerulenin	57	391	9	6007	0.003105336	0.005646066
mastoparan	10	206	2	6054	0.003885136	0.005977133
niguldipine	4	611	2	6060	0.003767185	0.005977133
tomatine	9	238	2	6055	0.004206531	0.00600933
gliotoxin	24	124	2	6040	0.01234541	0.016460546
clotrimazole	26	70	1	6038	0.035718741	0.044648426

x represents the number of known targets for the SMP drawn from the sub-network associated with the SMP; k represents the number of genes in the sub-network associated with the SMP; m represents the number of known drug targets for the SMP in the background gene set; n represents the number of genes in the background gene set excluded the known drug targets for the SMP; *p*-value is calculated as 1-phyper(x, m, n, k), where phyper is a function used in R.

In the SMP associated networks, we observed many perturbed interactions (ASCP and ASDP) between known drug targets and other uncharacterized genes in drug response, allowing for potential drug target predictions. Comparing between known drug target genes and other genes, we found that, in response to SMPs, ASCP or ASDP interactions tend to occur among drug target genes. Therefore, we predicted candidate drug targets based on their connecting degree with known drug targets in SMP associated networks. The candidate genes listed in Table S2 were all connected to 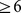 known drug targets. Among them, 59% (10/17) were found to exhibit resistance to the chemicals analysed in this study [41], [42], [43], [44], [45], [46], [47], [48], [49]. *INO4*, as an example, which is a transcription factor associated with phospholipids synthesis, shows differential interactions with nine genes targeted by eight drugs. Of these drugs, rapamycin, cycloheximide, lycorine, cerulenin, nocodazole, and resveratrol are classical cell proliferation inhibitors that are extensively utilized in cancer therapy and cell synchronization in molecular biology experiments [41], [42], [46], [47]. It has also been reported that gene *INO4* may be affected by bleomycin and fenpropimorph, and the mutant type will produce resistance. These two drugs are both antibiotics and have been considered for use in cancer treatment. *INO4* is seen as a potential drug target, particularly because it potentially plays a role in cell growth inhibition, and even cancer pathology. Regarding the other candidate genes, *RPN4*, *STE12*, *FKH2* and *CIN5* are also transcription factors that regulate important biological processes, such as RNA transcriptional elongation, *MAPK* signaling pathway, and proteasome genes expression. *YCK3*, *PKP2*, *PKP1*, and *SKM1* are important kinases and exhibit serine/threonine kinase, casein kinase and mitochondrial protein kinase activity. *FBA1* is fructose 1,6-biophosphate aldolase, which is required for glycolysis and gluconeogenesis. These genes exhibit certain functional features as drug targets, and published reports demonstrate that they exhibit reactions with certain known drugs and compounds; the natural or induced mutations will lead to mutant phenotypes. These annotations suggest that our genetic perturbing network results may be effective in drug target prediction.

Moreover, we conducted pathway enrichment analysis for each SMP associated network using DAVID based on KEGG. We detected 207 enrichment relationships (Beniamini<0.05) between 45 SMPs and 22 KEGG pathways (Figure S4). It has been demonstrated that multiple pathways are involved in a SMP response. This suggests that cooperation among pathways may exist in the process of small molecular drug responses and that there is the possibility of discovering new and non-intuitive relationships between biochemical pathways. We found that 124 pairs of pathways were involved in at least one common SMP response. Literature mining results based on PubMatrix [50] have provided strong independent evidence for the correlation among these pathways, 58% (72/124) of the pathway pairs co-occurred in at least one reference (detailed information for pathway pairs involved in >9 common SMPs is listed in Table S3). Some pathway pairs are already recognized as correlated pathways based on KEGG. Ten SMPs (cycloheximide, parthenlide, menadione and rapamycin et al.) clustered in the center of SMP-pathway graph (red rectangle area in Figure S4) targeted approximately the same pathways. These pathways all relate to protein biosynthesis, and are in concordance with the protein translation inhibition functional role of these SMPs. Some internal mechanisms of the SMP effects can be indicated in the SMP-pathway graph. For instance, glycolysis/gluconeogenesis is co-targeted by valinamycin, rapamycin, tunicamycin and fccp. However, the main effect of these four drugs is not regulation of the glucose distribution. A number of references suggest valinamycin plays a role in the glycolysis process by changing potassium concentration [51], rapamycin could influence the whole body glucose turnover [52], tunicamycin has the ability to regulate glycoprotein synthesis [53], and fccp could modulate the aerobic glycolysis by changing *P2X7* receptor activity [54]. A similar situation also appears in mismatch repair, proteasome, MAPK signalling pathway, etc. Interestingly, some biochemical pathways with non-intuitive relationships are targeted by common drugs such as pyruvate metabolism and steroid biosynthesis. Pyruvate metabolism and steroid biosynthesis seldom appear in the same physiological progress; however, in this study, we found that they are co-targeted by 6 SMPs. A study concerning feto-maternal metabolism suggested a high cholesterol consumption in the fetal compartment for cellular membrane synthesis and steroid biosynthesis, and the important intermediate process changes the blood pyruvate concentration and stimulates pyruvate metabolism [55]. This suggests that some new and possibly non-intuitive relationships between biochemical pathways may be observed based on our results.

Furthermore, in a previous study [56] it has been shown that genotyped markers can be used to predict the drug response (i.e., sensitivity or resistance) of the 104 individual genotyped yeast strains used in this study; they trained support vector machine (SVM) classifiers using 1, 10, 50, 100, 200, 500 and 1000 highest ranked marker(s) based on the linkage signals (lods value) between genotyped markers and SMP responses, and found the greatest marker-based prediction accuracy for each SMP response. In our study, for each SMP (at multiple time points and concentrations), we chose to use the markers perturbing its associated sub-network to perform the prediction, and obtained greater prediction accuracy for 65 SMP phenotypes (Figure S5). This finding indicates that the genetic variations that exhibit an effect on the molecular network state may be the more robust signal of phenotype variation.

**Figure 6:**
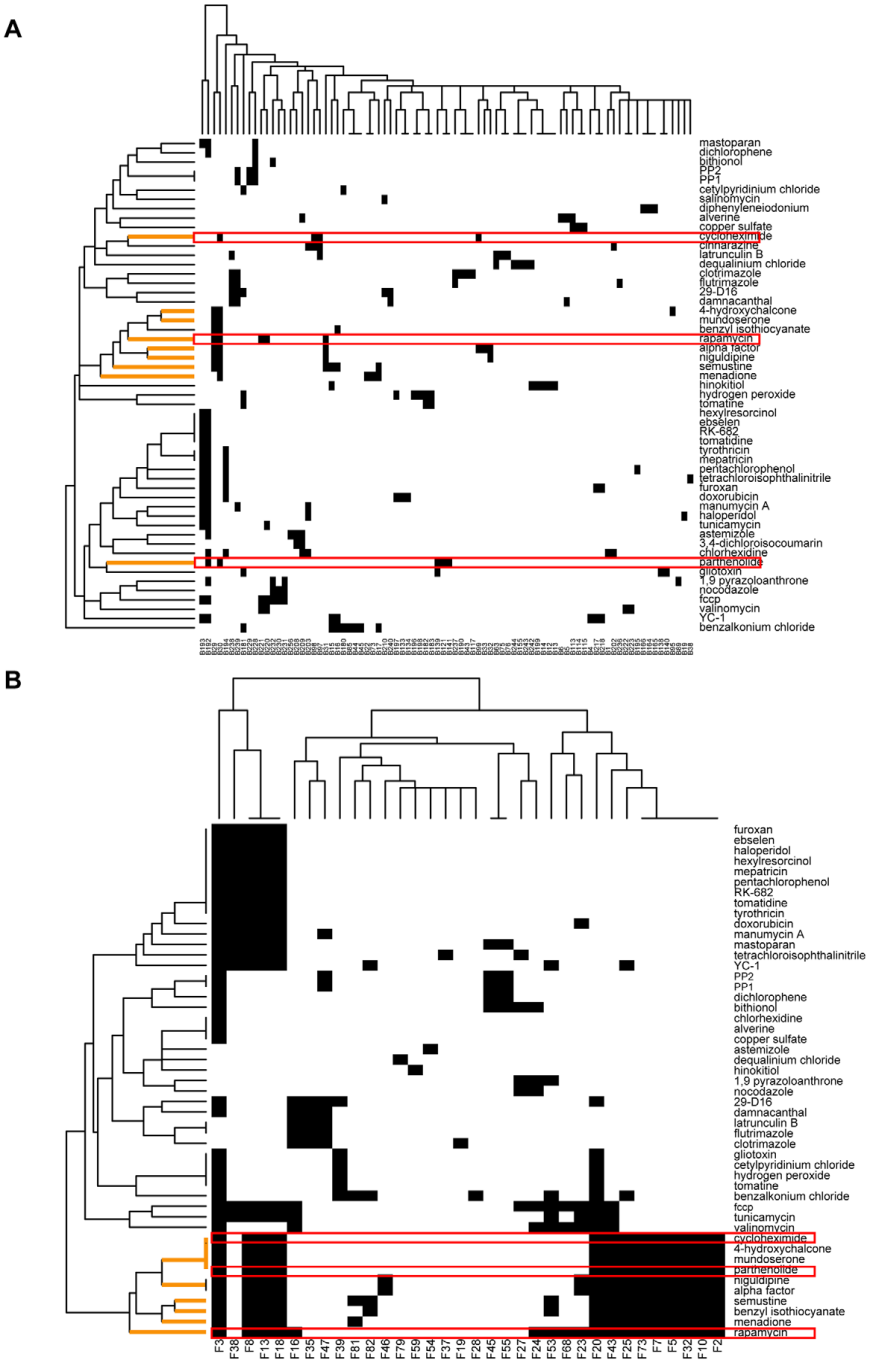
Comparison of the genetic and molecular basis of different SMP responses. (A) Hierarchical clustering of 53 selected SMPs shows clustering of genetic analogs. Columns represent 84 LD blocks; rows represent 53 SMPs. Black cell indicates that the QTLs located in the block are associated with the SMP. (B) Hierarchical clustering of 45 selected SMPs shows clustering of functional analogs. Columns represent 33 functional classes; rows represent 45 SMPs. Black cell indicates that the sub-network responding to the SMP is enriched in the functional class. SMP names are listed on the right-hand side of the clustergram.

We compared different SMPs responses; the QTLs associated with 53 SMPs were located in 84 block regions. As shown in Figure 6A, the two-dimensional hierarchical clustering result on the genetic association matrix reflects the genetic regulatory relationships between 84 blocks and 53 SMPs. We found that the response to SMP was a genetically complex trait, however, different SMPs primarily linked to specific chromosomal regions (blocks). Additionally, we performed functional enrichment analysis as described above for the sub-networks associated with 53 SMPs and we identified functional enrichment relationships between 45 SMPs and 33 functional classes. The two-dimensional hierarchical clustering (shown in Figure 6B) also indicated that different SMPs targeted particular functional classes. By comparing the two matrices, we found that certain SMPs associated with similar LD blocks usually shared common biological functions. For example, the SMPs indicated in orange clustered in Figure 6A are also highly correlated based on the functional association matrix in Figure 6B. Almost all of these seven compounds (aside from niguldipine) participate in the biological progress “inhibition of protein translation”, and they are all important drugs or precursor compounds in cancer treatment. Secondly, SMPs associated with different genetic blocks may also target common functional classes or pathways. For instance, rapamycin and cycloheximide, indicated by red rectangles in Figure 6A, only had one LD block in common, but targeted common biological processes such as carboxylic acid biosynthetic process, organic acid biosynthetic process, mitochondrial translation, nitrogen compound biosynthetic process, and protein serine/threonine kinase activity. These two SMPs are structurally unrelated and target different proteins, however, they both exhibit cell proliferation inhibition, and also similarly inhibit protein translation in cells [25] and are both important immunosuppressant drugs extensively used in organ transplantation [57], [58]. Taking rapamycin and parthenolide as another example, they are less common on a genetic basis, but in fact, they play a similar role in the biological context (induce cell apoptosis) and clinical effect (kill cancer cells) [57], [59] which is indicated more clearly in the functional association matrix (Figure 6B).

## Discussion

Networks and their variants provide an effective way to model biological systems and study their complex behavior. To understand the molecular and genetic basis of phenotypic variation, it is important to dissect the dynamic behavior of networks under genetic perturbations. In this study, we dissected the effects of genetic variations (SNPs) on an integrated interactome in yeast. Our results demonstrated that the interactome exhibited allele-specific behavior under genetic perturbations; the variants can be utilized to determine how genotypes affect small-molecule drug responses mediated by molecular networks in yeast.

In this study, in order to model how genetic variations are mediated by a network of molecular interactions in the cell, we proposed two types of allele-specific perturbations on interactions, termed ASCP and ASDP. In both cases, a genetic locus exerted a different influence on the transcriptional program of interactions for cases in which the locus exhibited distinct (alternative) alleles. ASCP and ASDP enable the assessment of variations at the genetic level that affect the expression of a gene and aid in the understanding of how these variations then alter the transcriptional program of interactions between the gene and its interactors in networks from two distinct aspects. In addition, we chose to analyze a hybrid interactome containing protein-protein, protein-DNA, kinase-protein, and enzyme-enzyme interactions. This allowed us to investigate several different mechanisms of action associated with genetic perturbations that normally occur because genetic perturbations may manifest through gains or losses of regulatory, signaling, and protein-complex interaction capabilities.

If the interactome is represented as a static network, more complex patterns of interactions that depend on temporal, spatial, or condition-specific contexts may be masked [60]. Rachlin et al. [60] reported the changing patterns of interactions from one biological context to another using a biological context network model. In our study, we demonstrated that the dynamic interactome exhibited a modular changing pattern under allele-specific context. Our findings suggest that the dynamic interactome can be viewed as a mosaic of overlapping sub-networks, each associated with an allele-specific context determined by a LD block. The allele-specific perturbation analysis that we introduce in this study connects chromosome regions (blocks) and network modules. Such modular structures may confer selective advantage by minimizing the impact of genetic variants outside of the module. The modules associated with the blocks exhibit significant functional roles, which is in concordance with the conclusion of Wu et al. [61] who demonstrated the association between eQTLs and functional gene sets. It is suggested that the possible functional role of these genetic variations could be mediated through the regulation of network modules. In addition, we observed that some regulator-pairs (LD block pairs), especially adjacent blocks co-regulated particular metabolic processes, signaling pathways or enzyme catalyzed processes (Figure 3). This may reflect the synergistic effect for combinations of loci in the regulation of common or correlated functions, which has some relevance with the epistatic interactions in a statistical genetic context.

In humans, many pharmacogenomics studies that assess the role of natural genetic variation in cellular response to small-molecule drugs are limited by small sample size and the inability to rapidly screen large numbers of drugs and phenotypes [62], [63]. Previous studies have shown that naturally recombinant yeast strains provide a suitable model for the study of therapeutically relevant complex traits (i.e, small-molecule drug response) [25], [64], [65] and may also serve as a model for personalized medicine [56]. In this study, we used a panel of 104 yeast strains designed to screen the drug response of 100 diverse small molecules in parallel. This method potentially aids in the discovery of common and specific mechanisms underlying various small molecule drugs. Moreover, our novel approach seeks to identify molecular network components that respond in trans to the genetic loci that drive variations in drug response, unlike the classic genetics approach that assesses the role of natural genetic variation in the cellular response to small-molecule drugs by identifying candidate genes underlying genetic loci. Literature mining results have provided strong independent evidence for the effectiveness of our allele-specific perturbing networks in the elucidation of small-molecule responses in yeast.

## Materials and Methods

### Data

Microarray and genotype data sets were obtained from a genome-wide eQTL study in yeast by Brem and Kruglyak [6] consisting of whole genome expression data for 112 yeast strains genotyped across 2956 genetic markers. Missing genotype data were imputed using a standard hidden Markov model algorithm implemented in R/qtl [66]. Missing expression data were imputed using the K = 15 nearest neighbors method [67].

We assembled a hybrid network of yeast from large-scale high-throughput screens and several interaction databases, totaling 66,569 unique pairwise interactions (160,730 non-unique interactions) among 6,064 proteins (genes), which contained 25,301 protein-protein interactions (the two proteins a and b display physical binding) obtained from the DIP database (Scere20101010) [68], 12681 protein-DNA regulatory interactions (a binds upstream of the gene encoding b) obtained from Beyer et al. (with log-likelihood score>4) [69], 28,785 phosphorylation events (a is a kinase that phosphorylates b) obtained from the Yeast Kinase Interaction Database (KID) [70], and 3,486 enzyme-enzyme metabolic relationships linked by common metabolites (a and b are enzymes that operate on at least one common metabolite) extracted from KEGG [71].

### Heritability Estimations

Heritability was calculated using the formula:
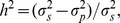



In which 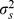 and 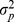 stand for the variance among phenotype values in the segregants and the pooled variance among parents, respectively [6]. We determined the significance of heritabilities via permutation: for each transcript, we combined all BY, RM, and segregant trait values, and then reassigned values to null parents and null segregants at random from this pool, and the 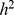 statistics were recomputed. Therefore, for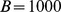 permutations of the trait values we obtained a set of null statistics 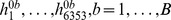. The *P* value for 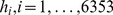 was calculated as:
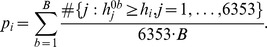



FDRs were computed according to [72], and the FDR = 0.05 cutoff was 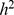
>0.669.

### Genome-Wide eQTL Mapping and SMP Response QTL Mapping

Because there are only two different alleles at each SNP locus in yeast (0 and 1), we tested the linkage between a marker and a transcript by partitioning the segregants into two groups according to marker genotype (0 or 1) and comparing the expression levels between the groups with the two-sample *t* statistic. The *t* statistic for the 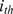 marker and the 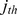 transcript is noted as 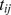.

We assessed significance via permutations. Specifically, the group labels on the segregants were randomly scrambled, and the *t* statistics were recomputed. Therefore, for 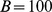 permutations of the array labels we obtained a set of null statistics 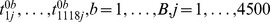. The *P* value for 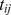 was calculated as:
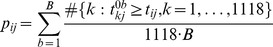



To adjust for multiple tests, we used FDR = 0.05 cutoff which corresponds to a *P*-value cutoff of 2.5×10E-4. Similarly, we used the t-test and permutation to detect QTLs for SMP response traits.

### Identify Allele-specific Perturbation of Interaction

**Figure 7:**
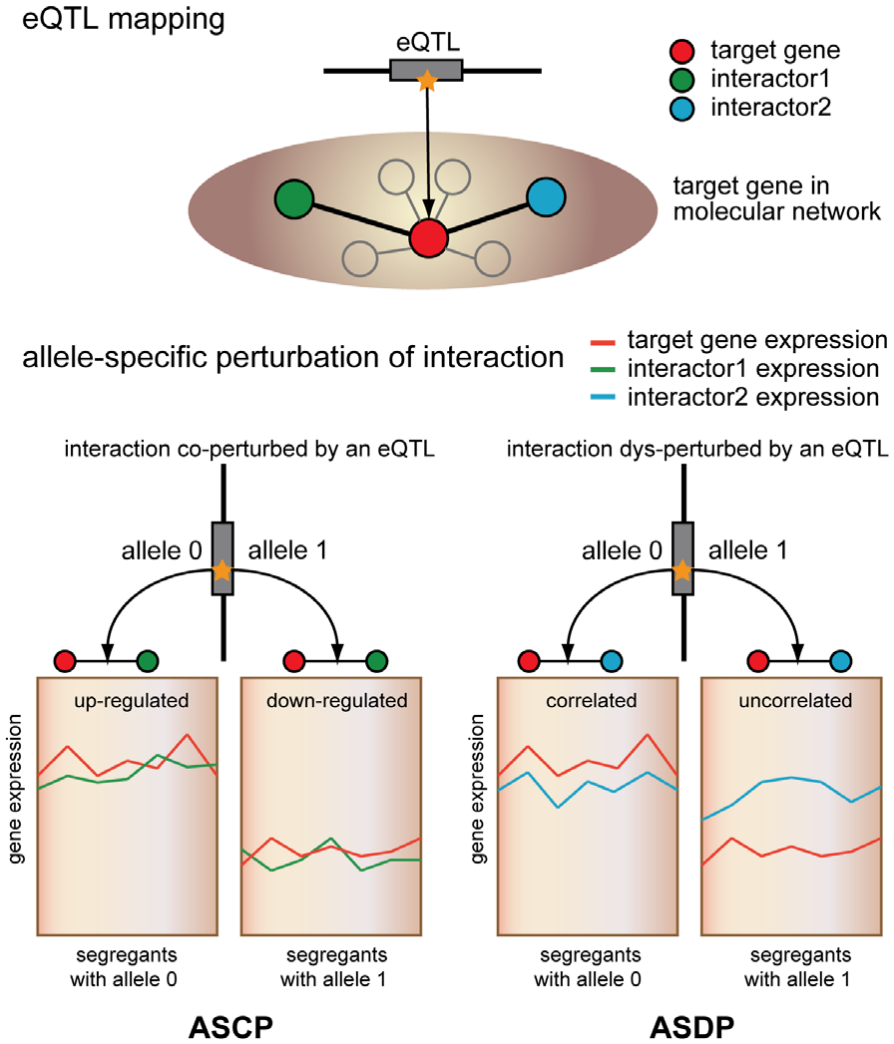
A schematic representation of two models of allele-specific perturbation of interaction: ASCP (left) and ASDP (right).

A schematic representation of the two models of allele-specific perturbation on interaction we defined in this study is provided in Figure 7. In one case, two interacting genes in the network were both targeted by an eQTL. We called this an allele-specific co-perturbation of interaction (ASCP) associated with the eQTL. In the other case, two interacting genes exhibited a significant difference in correlation of expression under different genotype groups of an eQTL. We called this type an allele-specific dys-perturbation of interaction (ASDP) associated with the eQTL.

In the left model, ASCP interactions are detected based upon the results of eQTL mapping above. ASCP indicates that two interacting genes both exhibit differential expression under the regulation of an eQTL. In the right model, the segregants are divided into two groups according to a specific eQTL genotype, because there are only two different alleles at each SNP locus in yeast (0 and 1). Next, we calculated the change in Pearson correlation coefficient of the expression of the two interacting genes according to the following equation:
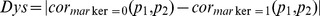
where 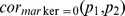
and 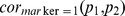 denote the Pearson correlation coefficient for expression vector of a target gene (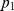) targeted by the eQTL and its network interactor (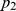) in the subgroup of samples with an allele 0 and an allele 1 at the specific SNP locus or eQTL, respectively. Thus, we obtain an estimate of the degree of dys-perturbation of an interaction by a SNP allele. To determine whether deviation in expression correlation between the two SNP allele groups is significant, we randomly reassigned the segregants to the two groups 1000 times and recalculated the Dys. Therefore, the *P*-value for ASDP of an interaction by an SNP or eQTL was given as the frequency of the values of the random Dys being greater than the value of the real Dys divided by 1,000. We controlled for multiple hypotheses using the false discovery rate, and only pairs with FDR over 0.05 were considered significantly ASDP.

### Randomization Test

To generate random networks that preserve the degree distribution of the input network, we utilized the integrated network as a seed network, and we then selected two edges at random and replaced them by two new edges (by random edge swapping), as described by Malsov and Sneppen [73]. We then repeated this until every edge in the network was rewired an average of 100 times. A total of 1000 randomized networks were generated using the above procedure to determine the *Z*-score and *p* value.

### Component Overlap Measure

To measure the overlap between two network components we defined a score termed the Component Share Ratio (CSR). The CSR score is calculated as the number of genes that occur in the intersection set of the two network components divided by the union of the two components.
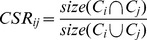



where 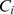 and 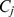 denote network components regulated by the 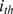 block and the 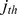 block, respectively.

### Enrichment Analysis of Known Drug Targets

We used hypergeometric distribution to test the enrichment of known drug targets in a SMP response associated sub-network, using the following formula:
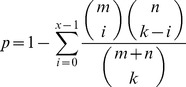
where *x* represents the number of known targets for the SMP drawn from the sub-network associated with the SMP; *k* represents the number of genes in the sub-network associated with the SMP; *m* represents the number of known drug targets for the SMP in the background gene set; *n* represents the number of genes in the background gene set excluded the known drug targets for the SMP.

## Supporting Information

Figure S1 **Global properties of the ASCP and ASDP effect on networks.** (A) The line chart shows the ratio tendencies of ASCP and ASDP in various types of interactions including PPI, PDI, KPI, EEI, and other particular types (PPIs belonging to the protein complexes; transcriptional regulation relations between TFs, TF-TF; phosphorylation events between kinase, K-K). The results indicate that the ratio of ASCP and ASDP exhibits significant differences in particular types of interactions compared to the four basic types of interactions. (B) CPN and DPN are generated by assembling all the ASCP and ASDP interactions, respectively. The CPN consists of 2184 ASCP interactions among 1375 genes and the DPN consists of 2416 ASDP interactions among 1821 genes. The different colors of edges represent different interaction types: PPI (green), PDI (red), KPI (blue), EEI (orange). (C) Degree distribution of the CPN and DPN. The examination of the degree distribution of both CPN and DPN reveals a power-law with a slope of −0.392 and R2 = ∼0.85 and a slope of −0.37 and R2 = ∼0.89 respectively. (D) Venn diagrams show the number of nodes (large intersection) and interactions (small intersection) that overlap between CPN and DPN.
(TIF)

Figure S2 **Hierarchical clustering on the association matrix between blocks and their perturbed interactions in the integrated network.** Rows represent interactions and columns represent blocks. Blocks associated with less than ten interactions are filtered out.
(TIF)

Figure S3 **A bipartite graph between 87 blocks and 48 functional groups, with triangles representing blocks and ovals representing functional groups.** A total of 57 relationships (between 36 blocks and 16 functional groups) marked red are screened out, because each of the 36 blocks perturbed more than five interactions among genes belonging to the same functional group. The 16 functional groups are marked with different colors.
(TIF)

Figure S4 **207 enrichment relationships (Beniamini<0.05) between 45 SMPs and 22 KEGG pathways.** Rectangles represent pathways, while diamonds represent SMPs.
(TIF)

Figure S5 **Comparison of the marker-based prediction accuracy of SMP responses.** Blue dots present the greatest marker-based prediction accuracies for SMP responses calculated using 1, 10, 50, 100, 200, 500 and 1000 highest ranked marker(s) to train the SVM in a previous study [56]. Red dots present the marker-based prediction accuracies calculated in this study, using the makers perturbing the SMP associated sub-networks to train the SVM.
(TIF)

Table S1 **Cis-acting or trans-acting regulation models between the genetic loci and their perturbed ASCP and ASDP interactions.** ‘Same chr’ denotes that two interacting genes are located on the same chromosomes, while ‘Diff chr’ denotes that two interacting genes are located on separate chromosomes. The value in each cell denotes the number of ASCP or ASDP interactions belonging to the particular category.
(DOC)

Table S2 **Candidate drug target genes.**
(DOC)

Table S3 **The pathway pairs involved in more than 9 common SMPs.** The third column denotes the number of common SMPs that a pathway pair is involved in. The fourth column represents the number of references that a pathway pair co-occurred. The fifth column represents the ratio value which is calculated as the frequency of a pathway pair co-occurrence/the frequency of an individual pathway occurrence in PubMed. The pathway pairs appearing in bold and italics are already known as correlated pathways based on KEGG.
(DOC)
