# Supplementary figures and images for: Allele-Specific Behavior of Molecular Networks: Understanding Small-Molecule Drug Response in Yeast
Source: PLoS One. 2013 Jan 4;8(1):e53581. doi: 10.1371/journal.pone.0053581 (PMC3537669; doi:10.1371/journal.pone.0053581)

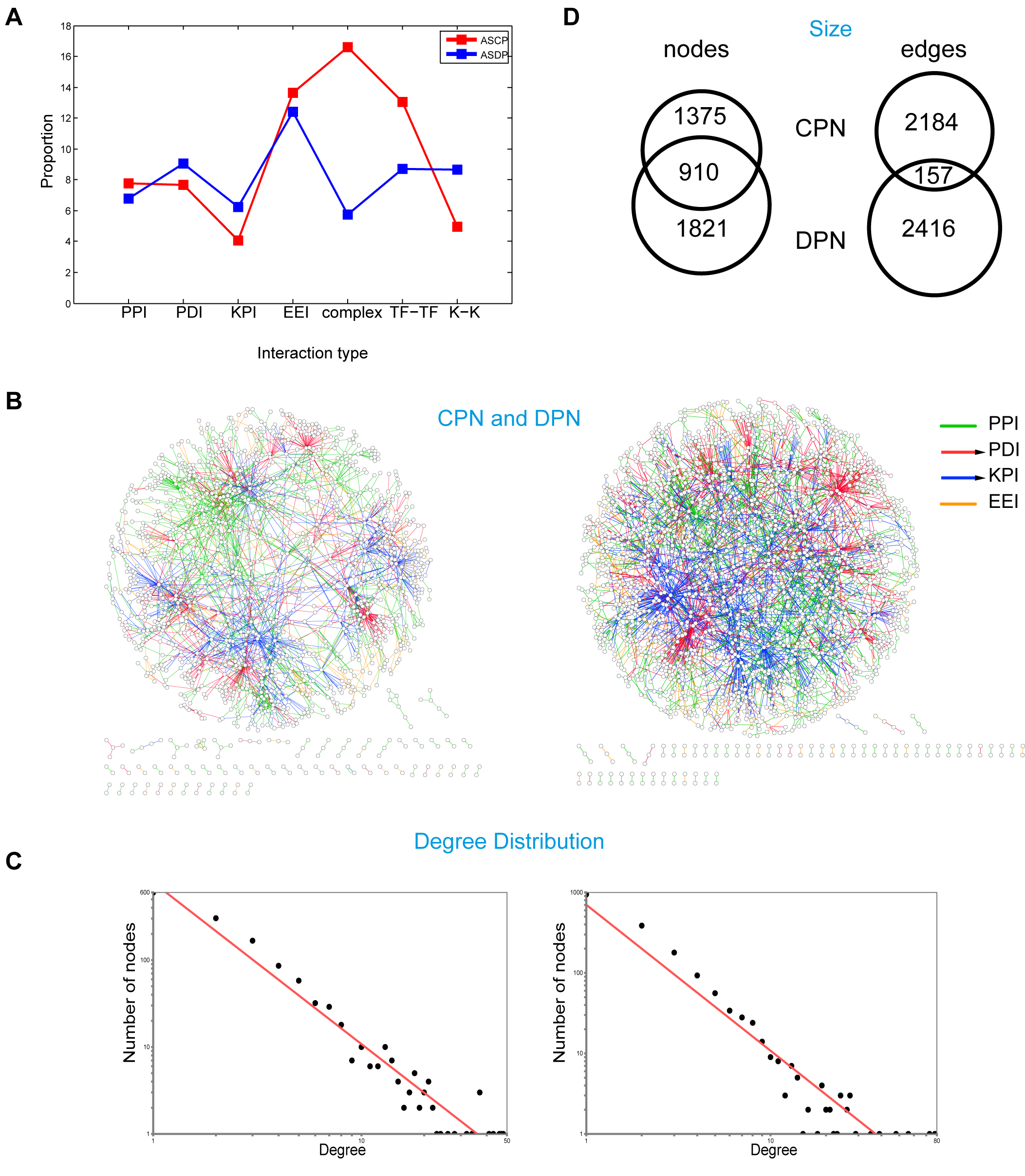

Supplement: Figure S1 — Global properties of the ASCP and ASDP effect on networks. (A) The line chart shows the ratio tendencies of ASCP and ASDP in various types of interactions including PPI, PDI, KPI, EEI, and other particular types (PPIs belonging to the protein complexes; transcriptional regulation relations between TFs, TF-TF; phosphorylation events between kinase, K-K). The results indicate that the ratio of ASCP and ASDP exhibits significant differences in particular types of interactions compared to the four basic types of interactions. (B) CPN and DPN are generated by assembling all the ASCP and ASDP interactions, respectively. The CPN consists of 2184 ASCP interactions among 1375 genes and the DPN consists of 2416 ASDP interactions among 1821 genes. The different colors of edges represent different interaction types: PPI (green), PDI (red), KPI (blue), EEI (orange). (C) Degree distribution of the CPN and DPN. The examination of the degree distribution of both CPN and DPN reveals a power-law with a slope of −0.392 and R2 = ∼0.85 and a slope of −0.37 and R2 = ∼0.89 respectively. (D) Venn diagrams show the number of nodes (large intersection) and interactions (small intersection) that overlap between CPN and DPN. (TIF) [file pone.0053581.s001.tif]

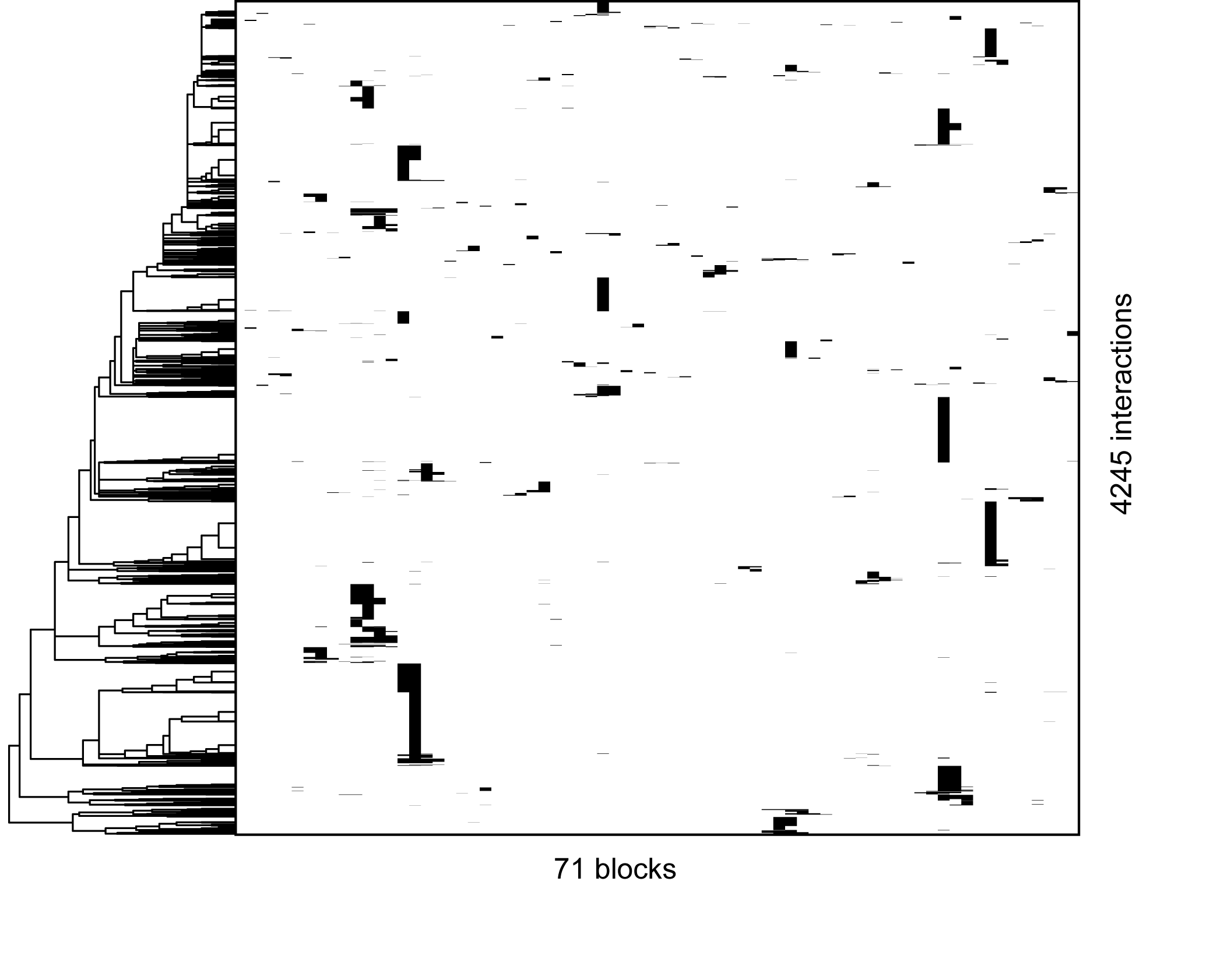

Supplement: Figure S2 — Hierarchical clustering on the association matrix between blocks and their perturbed interactions in the integrated network. Rows represent interactions and columns represent blocks. Blocks associated with less than ten interactions are filtered out. (TIF) [file pone.0053581.s002.tif]

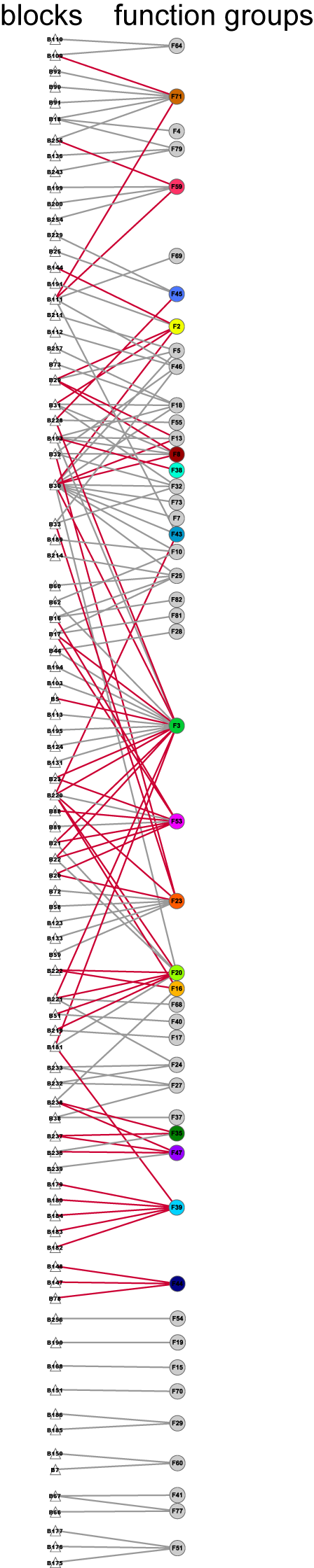

Supplement: Figure S3 — A bipartite graph between 87 blocks and 48 functional groups, with triangles representing blocks and ovals representing functional groups. A total of 57 relationships (between 36 blocks and 16 functional groups) marked red are screened out, because each of the 36 blocks perturbed more than five interactions among genes belonging to the same functional group. The 16 functional groups are marked with different colors. (TIF) [file pone.0053581.s003.tif]

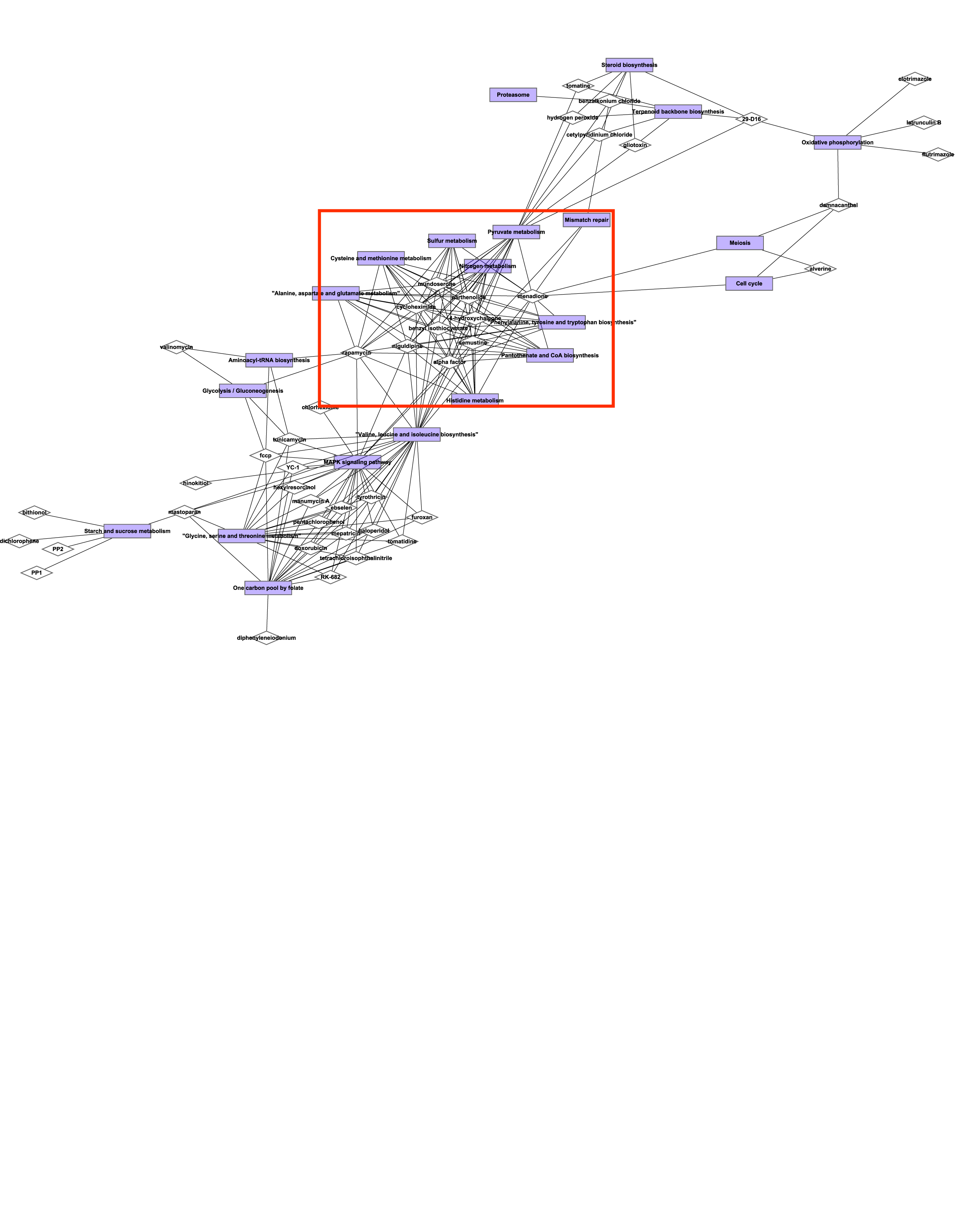

Supplement: Figure S4 — 207 enrichment relationships (Beniamini<0.05) between 45 SMPs and 22 KEGG pathways. Rectangles represent pathways, while diamonds represent SMPs. (TIF) [file pone.0053581.s004.tif]

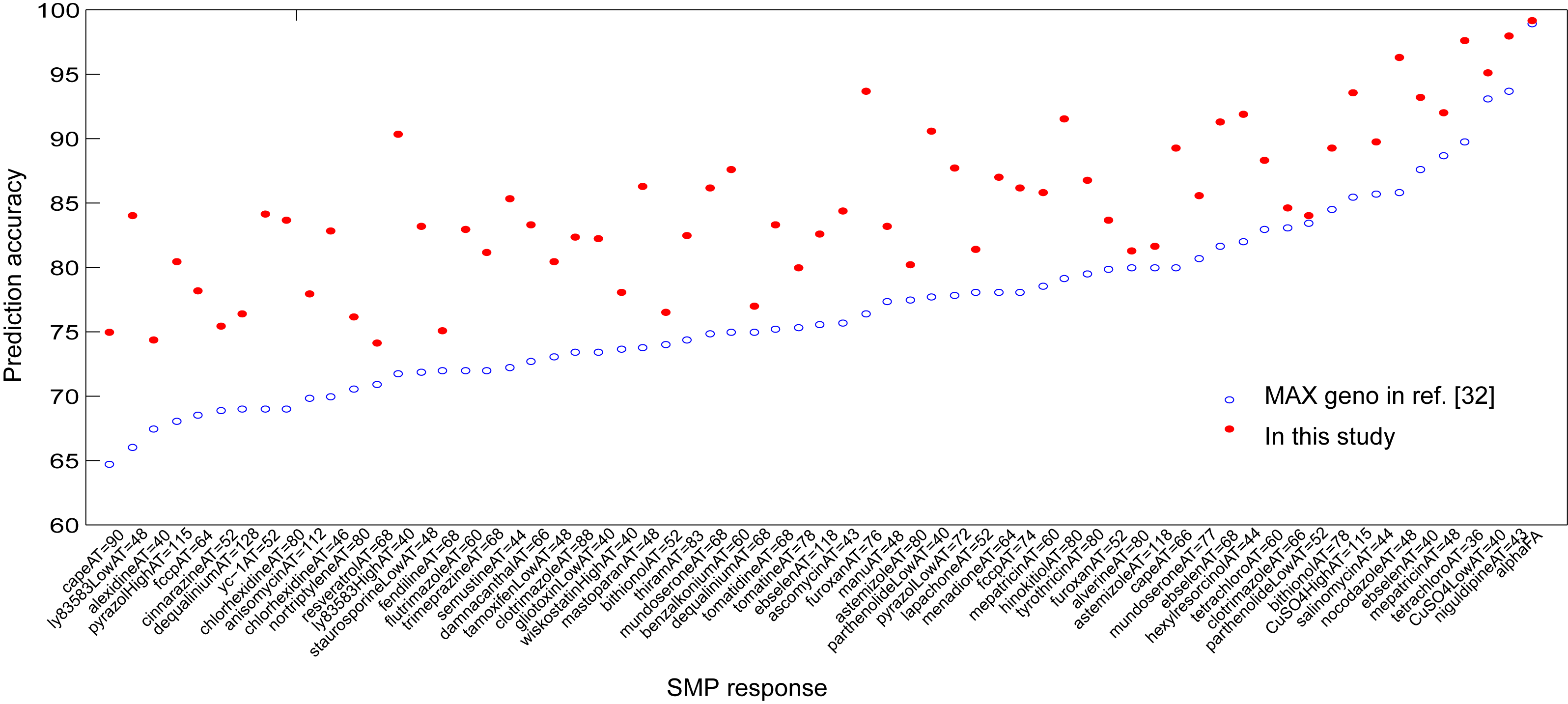

Supplement: Figure S5 — Comparison of the marker-based prediction accuracy of SMP responses. Blue dots present the greatest marker-based prediction accuracies for SMP responses calculated using 1, 10, 50, 100, 200, 500 and 1000 highest ranked marker(s) to train the SVM in a previous study [56]. Red dots present the marker-based prediction accuracies calculated in this study, using the makers perturbing the SMP associated sub-networks to train the SVM. (TIF) [file pone.0053581.s005.tif]
