# Supplementary material for: Allele-Specific Behavior of Molecular Networks: Understanding Small-Molecule Drug Response in Yeast
Source: PLoS One. 2013 Jan 4;8(1):e53581. doi: 10.1371/journal.pone.0053581 (PMC3537669; doi:10.1371/journal.pone.0053581)
Supplement: Table S1 — Cis-acting or trans-acting regulation models between the genetic loci and their perturbed ASCP and ASDP interactions. ‘Same chr’ denotes that two interacting genes are located on the same chromosomes, while ‘Diff chr’ denotes that two interacting genes are located on separate chromosomes. The value in each cell denotes the number of ASCP or ASDP interactions belonging to the particular category. (DOC) [file pone.0053581.s006.doc]

**Table S1. Cis-acting or trans-acting regulation models between the genetic loci** and their perturbed ASCP and ASDP interactions.

|  | Condition1 | Condition2 | Condition3 | Condition4 | Condition5 |
| --- | --- | --- | --- | --- | --- |
|  | 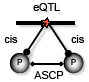 | 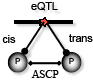 | 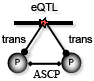 | 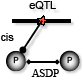 | 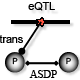 |
| total | 6 | 151 | 2167 | 273 | 2313 |
| Same chr | 6 | 151 | 186 | 36 | 194 |
| Diff chr | 0 | 0 | 1981 | 237 | 2119 |

‘Same chr’ denotes that two interacting genes are located on the same chromosomes, while ‘Diff chr’ denotes that two interacting genes are located on separate chromosomes. The value in each cell denotes the number of ASCP or ASDP interactions belonging to the particular [category](app:ds:category).
