# Supplementary material for: Allele-Specific Behavior of Molecular Networks: Understanding Small-Molecule Drug Response in Yeast
Source: PLoS One. 2013 Jan 4;8(1):e53581. doi: 10.1371/journal.pone.0053581 (PMC3537669; doi:10.1371/journal.pone.0053581)
Supplement: Table S2 — Candidate drug target genes. (DOC) [file pone.0053581.s007.doc]

**Table S2** Candidate drug target genes.

| Gene symbol | Gene title | Chemicals | References | Degree |
| --- | --- | --- | --- | --- |
| PKP2 (YGL059W) | Mitochondrial protein kinase that negatively regulates activity of the pyruvate dehydrogenase complex by phosphorylating the ser-133 residue of the Pda1p subunit; acts in concert with kinase Pkp1p and phosphatases Ptc5p and Ptc6p | resistance to chemicals: cycloheximide (45 ng/ml) | Alamgir et al., 2010, BMC Chem Biol, 10: p. 6 | 18 |
| CIN5 (YOR028C) | Basic leucine zipper (bZIP) transcription factor of the yAP-1 family; physically interacts with the Tup1-Cyc8 complex and recruits Tup1p to its targets; mediates pleiotropic drug resistance and salt tolerance; nuclearly localized under oxidative stress and sequestered in the cytoplasm by Lot6p under reducing conditions |  |  | 15 |
| YCK3 (YER123W) | Palmitoylated, vacuolar membrane-localized casein kinase I isoform; negatively regulates vacuole fusion during hypertonic stress via phosphorylation of Vps41p; shares essential functions with Hrr25p; regulates vesicle fusion in AP-3 pathway | resistance to chemicals: cycloheximide (45 ng/ml) | Alamgir et al., 2010, BMC Chem Biol, 10: p. 6 | 14 |
| FBA1 (YKL060C) | Fructose 1,6-bisphosphate aldolase, required for glycolysis and gluconeogenesis; catalyzes conversion of fructose 1,6 bisphosphate to glyceraldehyde-3-P and dihydroxyacetone-P; locates to mitochondrial outer surface upon oxidative stress |  |  | 13 |
| RPN4 (YDL020C) | Transcription factor that stimulates expression of proteasome genes; Rpn4p levels are in turn regulated by the 26S proteasome in a negative feedback control mechanism; RPN4 is transcriptionally regulated by various stress responses | resistance to chemicals:  cycloheximide, tunicamycin and rapamycin | Owsianik et al., 2002, Mol Microbiol, **43**(5): p. 1295-308;  Dudley et al., 2005, Mol Syst Biol, **1**: p. 2005 0001; Parsons et al., 2004, Nat Biotechnol, **22**(1): p. 62-9 | 13 |
| SKM1 (YOL113W) | Member of the PAK family of serine/threonine protein kinases with similarity to Ste20p and Cla4p; involved in down-regulation of sterol uptake; proposed to be a downstream effector of Cdc42p during polarized growth | resistance to chemicals:  hydrogen chloride (0.24% (w/v), pH 2.6) | Kawahata et al., 2006, FEMS Yeast Res, 6(6): p. 924-36 | 12 |
| FKH2 (YNL068C) | Forkhead family transcription factor with a major role in the expression of G2/M phase genes; positively regulates transcriptional elongation; negative role in chromatin silencing at HML and HMR; substrate of the Cdc28p/Clb5p kinase | resistance to chemicals: doxorubicin (50 ug/ml) | Westmoreland et al., 2009, PLoS One, 4(6): p. e5830 | 11 |
| PKP1 (YIL042C) | Mitochondrial protein kinase involved in negative regulation of pyruvate dehydrogenase complex activity by phosphorylating the ser-133 residue of the Pda1p subunit; acts in concert with kinase Pkp2p and phosphatases Ptc5p and Ptc6p | resistance to chemicals: cycloheximide (45 ng/ml) | Alamgir et al., 2010, BMC Chem Biol, 10: p. 6 | 10 |
| INO4 (YOL108C) | Transcription factor required for derepression of inositol-choline-regulated genes involved in phospholipid synthesis; forms a complex, with Ino2p, that binds the inositol-choline-responsive element through a basic helix-loop-helix domain | resistance to chemicals: rapamycin | Kapitzky et al., 2010, Mol Syst Biol, 6: p. 451 | 9 |
| STE12 (YHR084W) | Transcription factor that is activated by a MAP kinase signaling cascade, activates genes involved in mating or pseudohyphal/invasive growth pathways; cooperates with Tec1p transcription factor to regulate genes specific for invasive growth | resistance to chemicals: rapamycin | Xie et al., 2005, Proc Natl Acad Sci, 102(20): p. 7215-20 | 9 |
| GPM1 (YKL152C) | Tetrameric phosphoglycerate mutase, mediates the conversion of 3-phosphoglycerate to 2-phosphoglycerate during glycolysis and the reverse reaction during gluconeogenesis |  |  | 9 |
| SKP2 (YNL311C) | F-box protein of unknown function predicted to be part of an SCF ubiquitin protease complex; involved in regulating protein levels of sulfur metabolism enzymes; may interact with ribosomes, based on co-purification experiments | resistance to chemicals: [methylmercury chloride](http://www.yeastgenome.org/cgi-bin/phenotype/phenotype.fpl?rm=specific_tables&phenotype=resistance to chemicals: increased&property_value=methylmercury chloride) | Hwang et al., 2006, FEBS Lett, 580(30): p. 6813-8 | 9 |
| TEC1 (YBR083W) | Transcription factor targeting filamentation genes and Ty1 expression; Ste12p activation of most filamentation gene promoters depends on Tec1p and Tec1p transcriptional activity is dependent on its association with Ste12p; binds to TCS elements upstream of filamentation genes, which are regulated by Tec1p/Ste12p/Dig1p complex; competes with Dig2p for binding to Ste12p/Dig1p; positive regulator of chronological life span; TEA/ATTS DNA-binding domain family member |  |  | 8 |
| SWI6 (YLR182W) | Transcription cofactor; forms complexes with Swi4p and Mbp1p to regulate transcription at the G1/S transition; involved in meiotic gene expression; also binds Stb1p to regulate transcription at START; cell wall stress induces phosphorylation by Mpk1p, which regulates Swi6p localization; required for the unfolded protein response, independently of its known transcriptional coactivators | resistance to chemicals: tunicamycin and doxorubicin | Westmoreland et al., 2009, PLoS One, 4(6): p. e5830; Tan et al., 2009, Mol Biol Cell, **20**(5): p. 1493-508 | 7 |
| PGM2 (YMR105C) | Phosphoglucomutase, catalyzes the conversion from glucose-1-phosphate to glucose-6-phosphate, which is a key step in hexose metabolism; functions as the acceptor for a Glc-phosphotransferase |  |  | 7 |
| TDA1 (YMR291W) | Protein kinase of unknown cellular role; green fluorescent protein (GFP)-fusion protein localizes to the cytoplasm and nucleus; null mutant is sensitive to expression of the top1-T722A allele; not an essential gene |  |  | 7 |
| ENO1 (YGR254W) | Enolase I, a phosphopyruvate hydratase that catalyzes the conversion of 2-phosphoglycerate to phosphoenolpyruvate during glycolysis and the reverse reaction during gluconeogenesis; expression is repressed in response to glucose |  |  | 7 |
