# Supplementary material for: Allele-Specific Behavior of Molecular Networks: Understanding Small-Molecule Drug Response in Yeast
Source: PLoS One. 2013 Jan 4;8(1):e53581. doi: 10.1371/journal.pone.0053581 (PMC3537669; doi:10.1371/journal.pone.0053581)
Supplement: Table S3 — The pathway pairs involved in more than 9 common SMPs. The third column denotes the number of common SMPs that a pathway pair is involved in. The fourth column represents the number of references that a pathway pair co-occurred. The fifth column represents the ratio value which is calculated as the frequency of a pathway pair co-occurrence/the frequency of an individual pathway occurrence in PubMed. The pathway pairs appearing in bold and italics are already known as correlated pathways based on KEGG. (DOC) [file pone.0053581.s008.doc]

***Table S3 The pathway pairs involved in more than 9 common SMPs.***

| pathway1 | pathway2 | #SMPs | #refs | ratio |
| --- | --- | --- | --- | --- |
| ***Cysteine and methionine metabolism*** | ***Sulfur metabolism*** | ***10*** | ***1376*** | ***0.2534*** |
| Alanine, aspartate and glutamate metabolism | Pyruvate metabolism | 10 | 743 | 0.2101 |
| ***Glycine, serine and threonine metabolism*** | ***Valine, leucine and isoleucine biosynthesis*** | ***16*** | ***181*** | ***0.1451*** |
| ***Pantothenate and CoA biosynthesis*** | ***Pyruvate metabolism*** | ***10*** | ***2*** | ***0.1111*** |
| Phenylalanine, tyrosine and tryptophan biosynthesis | Valine, leucine and isoleucine biosynthesis | 10 | 209 | 0.0954 |
| ***Alanine, aspartate and glutamate metabolism*** | ***Histidine metabolism*** | ***10*** | ***326*** | ***0.0922*** |
| ***Alanine, aspartate and glutamate metabolism*** | ***Nitrogen metabolism*** | ***10*** | ***299*** | ***0.0846*** |
| Cysteine and methionine metabolism | Histidine metabolism | 10 | 428 | 0.0788 |
| Histidine metabolism | Nitrogen metabolism | 10 | 1654 | 0.0523 |
| Alanine, aspartate and glutamate metabolism | Valine, leucine and isoleucine biosynthesis | 10 | 184 | 0.052 |
| Cysteine and methionine metabolism | Nitrogen metabolism | 10 | 267 | 0.0492 |
| Phenylalanine, tyrosine and tryptophan biosynthesis | Pyruvate metabolism | 10 | 73 | 0.0333 |
| Nitrogen metabolism | Sulfur metabolism | 10 | 2570 | 0.0295 |
| Alanine, aspartate and glutamate metabolism | Cysteine and methionine metabolism | 10 | 85 | 0.024 |
| Cysteine and methionine metabolism | Valine, leucine and isoleucine biosynthesis | 10 | 130 | 0.0239 |
| Histidine metabolism | Sulfur metabolism | 10 | 560 | 0.0177 |
| Alanine, aspartate and glutamate metabolism | Phenylalanine, tyrosine and tryptophan biosynthesis | 10 | 62 | 0.0175 |

The third column denotes the number of common SMPs that a pathway pair is involved in. The fourth column represents the number of references that a pathway pair co-occurred. The fifth column represents the ratio value which is calculated as the frequency of a pathway pair co-occurrence/ the frequency of an individual pathway occurrence in PubMed. The pathway pairs appearing in bold and italics are already known as correlated pathways based on KEGG.
